# Structural and functional differences between bundles of different lengths: A corpus-driven study

**DOI:** 10.3389/fpsyg.2022.1061097

**Published:** 2023-01-05

**Authors:** Xuanjun Cui, Yoonjung Kim

**Affiliations:** Department of English Education, Kyungnam University, Changwon, Republic of Korea

**Keywords:** bundles, bundle lengths, structure, function, differences

## Abstract

This pilot study aims to investigate the differences between varying lengths of bundles in structure and function by comparing the 100 most frequent three-, four-, and five-word bundles in a self-built corpus of dissertations which contains about 3.5 million words. The findings reveal considerable variances between bundles of different lengths in terms of both structure and function. In general, the variances between three-and four-word bundles are greater than those between four-and five-word bundles, and three-and five-word bundles. Structurally, three-and four-word bundles differ significantly in all six main categories. Four-and five-word bundles vary in five categories, while three-and five-word bundles are only different in four categories. Functionally, noticeable variances were observed in research-, text- and participant-oriented bundles between three-and four-word bundles, and three-and five-word bundles. However, four-and five-word bundles only differ significantly in text- and participant-oriented bundles. Interestingly, bundles of varying lengths also vary in patterns that are used to perform the same functions. The results of this study might inform researchers that they need to take bundle lengths into consideration when making generalizations of their findings or comparing bundles between various studies.

## Introduction

Lexical bundles are “uninterrupted strings of three or more words that frequently recur in a register, identified empirically by running a computer program in a corpus of language texts” (Cortes, 2015, p. 205). They are also referred to as multiword sequences, multiword units, n-grams, lexical chunks, etc. The significance of bundles is reflected in the following aspects. Firstly, bundles are ubiquitous. Erman and Warren’s (2000) study suggests that formulaic sequences make up 58.6% of oral productions. This might be related to the fact that words tend to be used in the form of collocations (Szudarski, 2018). Secondly, bundles are stored in memory as whole so that they can be retrieved as whole when in use as well (Wray and Perkins, 2000). Therefore, a mastery of these bundles can help reduce processing time. In fact, this processing advantage is not only confined to native speakers. In their study, Conklin and Schmitt (2008) compared reading time for both formulaic and nonformulaic sequences by native and nonnative speakers, the findings indicated that both of them processed formulaic sequences more efficiently than nonformulaic ones. Thirdly, a mastery of these natural occurring bundles can assist L2 learners in producing more natural and native-like utterances; meanwhile, these bundles also help learners reduce collocational errors either in writing or speaking in that they can directly use them instead of combing single words or creating sequences creatively on their own (Lewis, 1993; Nesselhauf, 2005; Schmitt, 2010). Fourthly, there is an agreement that bundles are essential building blocks of coherent discourse (Biber et al., 2004; Hyland, 2008a; Biber, 2009; Schmitt, 2013; Ren, 2021). According to Hyland (2008b), bundles can shape meanings and help to make sense of coherence in a text. Ultimately, as different disciplines have divergent features in bundle use, a mastery of the frequent bundles in a certain field enables novice writers to quickly fit into the academic field that they belong to.

Additionally, the literature shows that bundles are also related to three aspects of language production, namely, complexity, accuracy, and fluency. There is robust evidence showing that bundles can facilitate fluency in production (Wood, 2010; Mohammadi and Enayati, 2018; Tavakoli and Uchihara, 2020; Nergis, 2021; Xuan et al., 2021). In Xuan et al.’s (2021) study, spoken fluency was measured in the aspects of articulation rate, speech rate, mean length of run, and phonation time ratio, the findings of which reveal a high correlation between bundle use and fluency. Meanwhile, studies (Lewis, 1993; Nesselhauf, 2005; Schmitt, 2010; Shin and Kim, 2017; Ma, 2020) also point to the positive role bundles play in accuracy. In Shin and Kim’s (2017) research, teaching articles with a bundle approach was proved to help students reduce their article omission errors. In contrast, the relationship between bundles and complexity is less obvious because existing studies seem to report contradictory results. Some studies (Mellow, 2006; Taguchi, 2008; Ma, 2020) found a positive effect of bundles on complexity, whereas others (Vercellotti et al., 2021) fail to observe such an effect.

Recent years have seen a growing interest in comparative studies into bundles. These studies tend to focus on the influence of text-source related variables on bundle use: registers (Biber and Barbieri, 2007; Biber, 2009), genres (Cock and Granger, 2021; Shirazizadeh and Amirfazlian, 2021), disciplines (Nekrasova-Beker and Becker, 2020; Cui and Kim, 2022), time (Hyland and Jiang, 2018; Candarli, 2021), or of author-related variables on bundle use: mother tongues (Esfandiari and Barbary, 2017; Pan et al., 2020), language proficiency (Chen and Baker, 2016; Siyanova-Chanturia and Spina, 2020), stay-abroad experience (Siyanova-Chanturia and Spina, 2020; Edmonds and Gudmestad, 2021). However, few studies are interested in exploring the effect of identification process-related variables on bundle use, such as frequency, range, and length. Evidently, this group of variables should not be neglected since they could determine what bundles we might get from a corpus, which then influence the distribution of various types of structure and function. The present study attempts to fill this gap by focusing on the variable of bundle length. Specifically, bundles of three different lengths were simultaneously extracted in the same corpus and comparisons were made between them to see whether they differ in terms of structure and function. It is notable that structural analysis was based on Biber et al.’s (1999) categorization, while functional analysis was based on Hyland’s (2008a) taxonomy. This is meaningful because most of previous studies (Chen and Baker, 2010; Esfandiari and Barbary, 2017; Lu and Deng, 2019; Shirazizadeh and Amirfazlian, 2021; Cui and Kim, 2022; Kim and Kessler, 2022) analyze bundles from two perspectives: structure and function. If bundles of different lengths differ in their structure and function, then the results of this study might have important implications for a wide range of bundle studies. For instance, researchers might need to indicate bundle length first when they make generalizations of their findings. In addition, they are supposed to compare bundles of the same length when contrasting bundles across studies. Otherwise, their generalizations or comparisons could be inappropriate and therefore unconvincing. Moreover, the frequent bundles identified in the study might benefit novice writers in the field of English education since they offer them a shortcut to quickly fit into the field. Furthermore, English for academic purposes (EAP) teachers might also find the bundles lists useful in that they can prioritize them while planning lessons. The present study was guided by the following research questions: (1) Do three-, four-, and five-word bundles differ in their distribution of structures? If yes, to what extent? (2) Do three-, four-, and five-word bundles differ in their distribution of functions? If yes, to what extent?

## Literature review

The past two decades have witnessed a surge of comparative studies into bundles, which can be broadly divided into two main lines according to their focus: (a) the influence of text-source related variables on lexical bundles: registers, genres, disciplines, time; and (b) the influence of author-related variables on lexical bundles: mother tongues, language proficiency, and stay-abroad experience.

### Text source-related variables

To begin with, studies focusing on the variable of registers thrived before 2010 (e.g., Conrad and Biber, 2005; Biber and Barbieri, 2007; Biber, 2009; Kim, 2009). For example, Conrad and Biber (2005) contrasted bundle use between academic and conversational prose. They found that the most frequent structural type in conversation is ‘personal pronoun + lexical VP (+ complement clause)’, which takes up 44% of the bundles, whereas that in academic prose is ‘preposition + NP fragment’, which makes up 33% of the bundles. Functionally, conversation prose is characterized by its high proportion of bundles that are used to express personal stance, while academic prose is characterized by its high proportion of referential bundles. Differences between the two even exist in the same bundle: ‘the fact that the’ in academic prose is used to convey certainty, but in conversation prose it is employed to express uncertainty.

Some researchers (Breeze, 2013; Cock and Granger, 2021; Shirazizadeh and Amirfazlian, 2021) concern the variable of genre. In Shirazizadeh and Amirfazlian’s (2021) study, bundle use in three different genres (i.e., textbooks, theses, articles) were compared, the results of which reveal significant differences between them. There is a significantly higher proportion of prepositional bundles in textbooks than in articles and theses. By contrast, articles and theses use a greater number of noun bundles than textbooks. Noticeable variances are also found in function: research-oriented bundles make up around 47% of the bundles in both articles and textbooks, but constitute roughly 60% of the bundles in theses. With regard to text-oriented bundles, only 27.7% of the bundles in theses fall into this category. However, the percentage of this category in articles and textbooks is 39.9 and 38.2%, respectively.

Other researchers (Hyland, 2008a; Hyland and Jiang, 2018; Omidian et al., 2018; Nekrasova-Beker and Becker, 2020) attempt to explore disciplinary variance of bundles. This is one of the most extensively explored variables within this line. Omidian et al. (2018) made a comparison between hard science fields (biology, physics, mechanical engineering) and soft science fields (sociology, marketing, applied linguistics) in their use of bundles. They found that writers in hard and soft disciplines have different focus: those in hard sciences prioritize their methodology, while those in soft sciences highlight their research goals and implications for future studies. Another difference is that writers in soft fields are more inclined to employ bundles to describe intangible attributes of research processes or procedures, whereas their counterparts in hard sciences use more bundles to explain the physical attributes of research objects. In addition, compared with hard fields, soft fields make use of more hedging devices (e.g., are more likely to) and summarize their outcomes in a less assured manner, which help to reduce the possibility of overgeneralization.

The last variable within this line is time. Studies of this category (Siyanova-Chanturia, 2015; Hyland and Jiang, 2018; Hong, 2019; Candarli, 2021; Vercellotti et al., 2021) investigate whether bundle use change over time. An exemplary study is Hyland and Jiang (2018) research which explored the changes of bundles in four disciplines from 1965 to 2015. It is found that electronic engineering has witnessed great changes over time: the proportion of verb phrase-related bundles has decreased from 34.5 to 26.8%, while that of noun/preposition-related bundles has increased from 45.7 to 56.2%. From the perspective of function, an increase can be observed in the proportion of research-oriented bundles in applied linguistics and sociology, yet a decrease was found in biology and electronic engineering. What is more, all fields have witnessed a proportional growth in text-oriented bundles except electronic engineering. Furthermore, there is a decline in the percentage of participant-oriented bundles in applied linguistics and sociology, yet a growth in biology and electronic engineering.

### Author-related variables

In additional to the text source-related variables mentioned above, researchers are also keen to investigate author-related variables. Within this line, many researchers (Esfandiari and Barbary, 2017; Liu and Lu, 2019; Lu and Deng, 2019; Cui and Kim, 2021a) are concerned with the differences in bundle use between learners with various L1 backgrounds. For instance, Cui and Kim (2021a) compared bundle use between L1 Korean and L1 Chinese writers in their English dissertations. The results are that Chinese writers generally make use of more bundles than Korean writers. Structurally, Korean writers use a higher proportion of noun-based and preposition-based bundles than their Chinese counterparts. By contrast, Chinese writers use a greater number of bundles falling into the category of “other bundles.” As for function, Korean writers employ a greater number of resultative and framing bundles, whereas Chinese writers use a higher proportion of transition and text-reflexive bundles. Chinese writers are also found to use bundles of different types more evenly in their dissertations. Interestingly, Chinese writers tend to underuse bundles while their Korean counterparts are inclined to overuse bundles.

Some researchers (Chen and Baker, 2010, 2016; Xu, 2012; Siyanova-Chanturia and Spina, 2020) are interested in revealing the relationship between language proficiency and bundles use. Chen and Baker (2016) contrasted bundles use in English essays by L1 Chinese learners of three different proficiency levels: CEFR B1, B2 and C1. They observed that C1 (the highest level) learners’ essays are the closest to academic prose in that they have the lowest proportion of VP-based bundles (44%) and the highest percentage of PP- and NP-based bundles. On the contrary, B1 (the lowest level) learners’ essays are the most similar to conversation prose because of their highest proportion of VP-based bundles (78%) and the lowest percentage of PP- and NP-based bundles. When it comes to function, C1 learners use a greater number of stance bundles and referential expressions than B1 and B2 learners. In comparison, B1 and B2 learners make use of more discourse organizers than C1 learners. Differences also exist in subcategories. For instance, within stance bundles, the proportion of epistemic type decreases as learners’ proficiency level increases, yet the combined percentage of attitudinal and modality types increases as learners’ proficiency level increases.

Others (Yoon, 2016; Siyanova-Chanturia and Spina, 2020; Edmonds and Gudmestad, 2021) are concerned with the influence of stay abroad experience on collocations. Edmonds and Gudmestad (2021) traced the development of learners’ ‘noun+adjective’ collocations in written assignments over a stay in a target language environment, the findings reveal that staying abroad experience contributes to an increase in the use of ‘noun + adjective’ combinations that consist of low frequency words. In another study conducted by Siyanova-Chanturia and Spina (2020) which involves 175 participants, the use of ‘noun + adjective’ collocations has witnessed an overall decrease after the staying abroad experience. At the same time, they also found that beginner learners are more inclined to use low frequency collocations after staying abroad. Surprisingly, instead of contributing to more idiomatic production, high proficiency and sufficient exposure to L2 are found to cause heavy reliance on less frequent combinations.

To summarize, it is not hard to find that most bundle studies tend to analyze bundles in terms of structure and function, and calculate the proportion of different categories of structure and function. Notably, while the two lines of studies mentioned above thrive, few attempts have been made to explore the influence of identification process-related variables on the structure and function of bundles, such as frequency, range, and length. These factors would inevitably exert an influence on what bundles we could extract from a corpus, which might then directly influence the calculation of the proportion of different structures and functions since different bundles differ in structure and function. Therefore, it is necessary to conduct empirical studies to reveal the effect of these variables on the structure and function of bundles. It is true that there are some studies which have covered varying lengths of bundles, yet they either merely focus on four-word bundles (e.g., Ren, 2021), or do not use the same criteria when extracting bundles of different lengths because bundle lengths is not their focus (e.g., Esfandiari and Barbary, 2017). Thus, a direct comparison between bundles of varying lengths in terms of structure and function is currently unavailable.

## Methodology

### Corpus building

As demonstrated in Table 1, the corpus developed in this study consists of 90 doctoral dissertations in English education which were downloaded from *ProQuest*. We chose 90 dissertations because it is large enough to identify the number of bundles that we need. When choosing doctoral dissertations, only three criteria were involved. The first is that they must be in the field of English education. The second is that they must be written in English. Thirdly, they were written between 1991 and 2020. Specifically, 30 dissertations were written in the period of 1991–2000, 30 were in 2001–2010, and 30 in 2011–2020. In other words, other factors like the writers’ L1 background, language proficiency, and where they study were not considered. The selected dissertations then went through a manual deletion process during which only main body of the dissertations was maintained, while other parts such as abstracts, acknowledgement and dedications, table of contents, references, titles, footnotes, charts, tables, etc. were all discarded. Finally, the remaining parts of all dissertations were compiled into a corpus of 3,585,637 words.

**Table 1:** Description of the corpus.

No. of texts	Total length (in words)	Average length (in words)
90	3,585,637	39840.4

### Identification process

Length, frequency and range are the three most significant criteria in the process of extracting bundles. As for length, previous studies tend to focus on three-, four-, and five-word bundles. Four-word bundles have received the most attention (Hong, 2019; Liu and Lu, 2019; Lu and Deng, 2019; Shirazizadeh and Amirfazlian, 2021; Yin and Li, 2021; Cui and Kim, 2022), followed by three-and five-word bundles. This study only involves three- to five-word bundles because the number of two-word bundles is too overwhelming, while six-word bundles or longer ones contain too many shorter bundles, which might cause overlapping issues. More importantly, three- to five-word bundles are the most studied ones, so a comparison of which might inform more studies. Based on the above reasons, this study only compares 3-, 4-, and 5-word bundles.

Also, setting a frequency threshold is an essential step for bundle identification. However, such a threshold can be arbitrary and there is little consensus as to what is the standard. It might vary from occurring at least 10 times per million words (Biber et al., 1999; Simpson-Vlach and Ellis, 2010), 20 times per million words (Cunningham, 2017; Hyland and Jiang, 2018; Lu and Deng, 2019; Shirazizadeh and Amirfazlian, 2021), to 40 times per million words (Biber and Barbieri, 2007; Pan et al., 2016) in a corpus. The criteria could change according to corpus size, research purposes, etc. A common conventional practice is to set it as appearing at least 20 times per million words. Following this convention, the current study adopted 20 times per million words as cut-off frequency.

Range is another crucial factor in bundle extraction. Previous studies tend to set it as appearing in at least 5 different texts (Biber et al., 2004; Pan et al., 2016; Omidian et al., 2018; Cui and Kim, 2021b), or in 10% of all the texts (Hyland, 2008a; Hong and Hua, 2018; Hyland and Jiang, 2018; Shirazizadeh and Amirfazlian, 2021; Yin and Li, 2021) in a corpus. Researchers might choose a range threshold out of different concerns, but the total number of texts is a main concern. Following the practice of previous research, the present study adopted an occurrence in 10% of texts as criterion.

AntConc, a software for corpus processing, was employed to identify three-, four-, and five-grams in the corpus. The criteria are that they must appear in 10% of all the dissertations with a minimum frequency of 20 times per million words. Then we obtained 8,487 three-grams, 1,375 four-grams, and 169 five-grams. All these bundles then went through a manual selection process to exclude the unqualified ones. For example, when checking four-word bundles, “in the u s” was abandoned because it is mistakenly taken by machine as four-grams. In the end, the top 100 remaining three-, four-, and five-grams were chosen to build a bundle list, respectively. Their total frequency are 53,991, 16,207, and 5,403 times, respectively.

### Data analysis

The qualified bundles, namely the bundles in the three lists, were then manually analyzed in terms of structure and function. The structural analysis was performed using the framework demonstrated in Table 2. This framework groups bundles into six main types: noun phrase-based bundles, preposition phrase-based bundles, verb phrase-based bundles, anticipatory it structure, infinitive construction, and “others.” The first two categories were furthered divided into subtypes due to their greater number. It is notable that this taxonomy was adapted from Biber et al.’s (1999, p. 997–1,025) structural framework which categorized four-word bundles into eight subcategories: prepositional phrase + of, other prepositional phrases, noun phrase + of, other noun phrases, passive + prep phrase fragment, anticipatory it +verb/adj, be + noun/adjectival phrase, and “others.” As the present study involves bundles of different lengths, substantial changes were made in the original framework so as to make it more suitable for this study. For example, the category of *passive + prep phrase fragment* was removed because few bundles in this study fall into it.

**Table 2:** Structural framework for bundle analysis.

Noun phrase-based bundles
NP with of-phrase fragment (e.g., a variety of, the nature of the)
Other NP (e.g., the target language, participants in this study)
Preposition phrase-based bundles
PP-based bundles beginning with in (e.g., in other words the)
PP-based bundles beginning with of (e.g., of the program)
PP-based bundles beginning with on (e.g., on the other hand the)
PP-based bundles beginning with at (e.g., at the secondary school level)
Other PP-based bundles (e.g., according to the, through the use of)
Verb-based bundles (e.g., related to the, was one of the, play an important role in)
Anticipatory it structure (e.g., it is important to, it should be noted that)
Infinitive construction (e.g., to learn English, to participate in the, to meet the needs of)
Others (e.g., as well as, culturally and linguistically diverse, and at the same time)

Functional analysis was performed based on Hyland’s (2008a) taxonomy. Hyland categorized bundles into three main types: text-oriented, research-oriented, and participant-oriented bundles. Text-oriented bundles focus on “the organization of the text and its meaning as a message or argument”; research-oriented bundles “help writers to organize their activities and experiences of the real world”; participant-oriented bundles center on “the writer or reader of the text” (p. 13–14). Specifically, text bundles contain four subcategories: transition bundles, structuring bundles, framing bundles, and resultative bundles. Research bundles comprise five subtypes: location bundles, topic bundles, description bundles, quantification bundles, as well as procedure bundles. And participant bundles can be further divided into stance bundles and engagement bundles. In the current study, bundles were categorized into three main types, namely research, text and participant bundles, without further dividing because of the relatively small number of five-word bundles.

Then the results of structural and functional analysis of bundles were compared between three-and four-word bundles, four-and five-word bundles, and three-and five-word bundles. Considering that the frequency of five-word bundles is significantly less than that of three-and four-word bundles, this study did not directly compare the frequency of bundles. Instead, the proportion of each structural and functional category was used for comparison. Meanwhile, log-likelihood test was performed to reveal the statistical differences between three- to five-word bundles.

## Results

The total frequency of the three-, four-, and five-word bundle lists are 53,991, 16,207, and 5,403 times, respectively. Three-word bundles occur roughly three times as frequent as four-word bundles, whereas four-word bundles appear about three times as frequent as five-word bundles. It is evident that shorter bundles occur much more frequently than longer ones. Detailed information concerning the three bundle lists can be found in Table 3.

**Table 3:** The top 50 three-, four-, and five-word bundles.

as well as (1641)	in the united states (680)	English as a second language (613)
in order to (1520)	as a second language (636)	English as a foreign language (173)
English language learners (1342)	English as a second (621)	at the end of the (159)
one of the (1290)	of English language learners (481)	to meet the needs of (134)
in the classroom (1132)	on the other hand (377)	the field of English education (124)
the united states (1121)	as a result of (324)	the purpose of this study (113)
English as a (1085)	the ministry of education (324)	national council of teachers of (109)
the use of (969)	as well as the (301)	council of teachers of English (103)
a second language (916)	the end of the (293)	of English language learners in (98)
in this study (912)	at the end of (287)	the results of this study (97)
of English language (894)	in the area of (277)	at the beginning of the (94)
of this study (864)	at the same time (274)	of this study was to (94)
of the study (855)	the results of the (259)	the national council of teachers (87)
in terms of (797)	in the field of (243)	a language other than English (79)
the English language (782)	to be able to (232)	English as an international language (79)
the teaching of (782)	the teaching of English (226)	as a result of the (78)
the importance of (761)	English language learners in (216)	purpose of this study was (76)
part of the (704)	as a foreign language (214)	the findings of this study (76)
English language arts (699)	one of the most (209)	an English as a second (69)
in the united (687)	the purpose of this (209)	the teaching of English in (68)
be able to (675)	it is important to (202)	in the united states and (65)
some of the (665)	meet the needs of (190)	culturally and linguistically diverse students (62)
as a result (660)	English as a foreign (183)	one of the most important (61)
as a second (657)	of the English language (182)	it should be noted that (59)
there is a (648)	the field of English (177)	the purpose of the study (59)
the purpose of (641)	the purpose of the (174)	the no child left behind (57)
based on the (615)	limited English proficient students (164)	on the part of the (55)
a lot of (590)	first year of teaching (155)	to participate in the study (54)
the development of (572)	at the beginning of (153)	and be able to do (53)
of the English (565)	the beginning of the (150)	in the field of English (53)
the number of (556)	in special education programs (149)	on the other hand the (50)
the needs of (542)	the role of the (148)	the common core state standards (50)
the role of (538)	is one of the (143)	by the end of the (49)
reading and writing (536)	the rest of the (142)	by the ministry of education (49)
based on their (534)	of English as a (140)	no child left behind act (46)
teaching and learning (533)	to meet the needs (138)	at the time of the (42)
students in the (525)	for English language learners (137)	elementary and secondary education act (40)
the end of (506)	of this study was (137)	play an important role in (40)
the field of (501)	the teaching of writing (136)	there is a need for (40)
of the students (494)	field of English education (133)	in the field of education (39)
in the field (490)	the English language arts (133)	at the high school level (38)
the results of (483)	in the case of (131)	for the purposes of this (38)
a variety of (479)	in the state of (131)	in the area of English (38)
according to the (469)	of teachers of English (131)	of the ministry of education (38)
in addition to (459)	in addition to the (130)	the elementary and secondary education (38)
of English education (457)	in the process of (130)	the role of the teacher (38)
teacher education programs (450)	on the part of (128)	in an English speaking country (37)
ministry of education (441)	in the form of (127)	the results of the study (37)
the fact that (441)	in the target language (125)	in the teaching of writing (36)
in special education (439)	for the purpose of (123)	is one of the most (36)

### Comparison between three-and four-word bundles

Structurally, log-likelihood test shows that significant differences exist between three-and four-word bundles in the distribution of all six main categories: NP-based bundles (log-likelihood = 344.99, *p* < 0.001***), PP-based bundles (log-likelihood = 790.19, *p* < 0.001***), VP-based bundles (log-likelihood = 147.90, *p* < 0.001***), anticipatory it structure (log-likelihood = 63.48, *p* < 0.001***), infinitive construction (log-likelihood = 8.40, *p* < 0.01**), and “others” (log-likelihood = 168.11, *p* < 0.001***). The most substantial variance between three-and four-word bundles was observed in the proportion of PP-based bundles. As shown in Table 4, 32.7% of three-word bundles are PP-based structures, but 48.4% of four-word bundles are of this category. What follows is the variance in NP-based bundles. While 46.5% of three-word bundles are categorized as NP-based patterns, only 35.7% of four-word are grouped into this category. Overall, three-word bundles have a higher percentage of NP-based bundles, VP-based bundles, and “others,” whereas four-word bundles contain a higher proportion of PP-based bundles, anticipatory it structure, and infinitive construction.

**Table 4:** Structural distribution of three-, four-, and five-word bundles.

Category	Subcategory	3-word		4-word		5-word	
Noun phrase-based bundles	NP with of-phrase fragment	13,394	24.8%	4,041	24.9%	1,193	22.1%
	Other NP	11,712	21.7%	1749	10.8%	1,221	22.6%
	Subtotal	25,106	46.5%	5,790	35.7%	2,414	44.7%
Preposition phrase-based bundles	PP-based bundles beginning with in	8,258	15.3%	2,976	18.4%	407	7.5%
	PP-based bundles beginning with of	5,863	10.9%	1,508	9.3%	376	7.0%
	PP-based bundles beginning with on	421	0.8%	505	3.1%	105	1.9%
	PP-based bundles beginning with at	641	1.2%	1,047	6.5%	447	8.3%
	Other PP-based bundles	2,490	4.6%	1814	11.2%	357	6.6%
	Subtotal	17,673	32.7%	7,850	48.4%	1,692	31.3%
Verb phrase-based bundles		2,552	4.7%	422	2.6%	138	2.6%
Anticipatory it structure		320	0.6%	202	1.2%	94	1.7%
Infinitive construction		1,390	2.6%	487	3.0%	240	4.4%
Others		6,950	12.9%	1,456	9.0%	825	15.3%
Grand Total		53,991	100%	16,207	100%	5,403	100%

In terms of subcategory, significant differences were found in all types except NP with of-phrase fragment. The most enormous difference was found in PP-based bundles beginning with at (log-likelihood = 1164.62, *p* < 0.001***). This subcategory only takes up 1.2% of three-word bundles, but make up 6.5% of four-word bundles. Considerable variances also exist in “other NP” (log-likelihood = 877.73, *p* < 0.001***) and “other PP-based bundles” (log-likelihood = 765.47, *p* < 0.001***). The proportion of “other NP” in four-word bundles is 10.8%, merely half of that in three-word bundles. In contrast, the proportion of “other PP-based bundles” in three-word bundles (4.6%) is less than half of that in four-word bundles (11.2%). Meanwhile, the variance in PP-based bundles beginning with on is also noticeable: it accounts for 3.1% of four-word bundles, yet merely comprises 0.8% of three-word bundles. Interestingly, the proportion of all subcategories of PP-based bundles in four-word bundles is higher than that in three-word bundles except the category of PP-based bundles beginning with of.

Functionally, there are significant differences between three-and four-word bundles in the distribution of research-oriented bundles (log-likelihood = 149.37, *p* < 0.001***), text-oriented bundles (log-likelihood = 215.36, *p* < 0.001***), and participant-oriented bundles (log-likelihood = 10.02, *p* < 0.01***). As shown in Table 5, the biggest difference between three-and four-word bundles lies in their proportion of text-oriented bundles. It ranges from 34.3% in three-word bundles to 42.4% in four-word bundles. Another considerable difference is in research-oriented bundles: its proportion varies from 63.6% in three-word bundles to 55.1% in four-word bundles. What is more, the distribution of different types of bundles is relatively more even in four-word bundles. In three-word bundles, the proportion of research-oriented bundles is close to double of that of text-oriented bundles, with participant-oriented bundles making up a particularly small proportion. However, in four-word bundles, the proportion of text- and participant-oriented bundles have both increased.

**Table 5:** Functional distribution of three-, four-, and five-word bundles.

Category	3-word		4-word		5-word	
Research-oriented	34,325	63.6%	8,929	55.1%	2,930	54.2%
Text-oriented	18,528	34.3%	6,867	42.4%	2073	38.4%
Participant-oriented	1,138	2.1%	411	2.5%	400	7.4%
Total	53,991	100%	16,207	100%	5,403	100%

### Comparison between four-and five-word bundles

Significant variances exist between four-and five-word bundles in the distribution of all main structural categories except VP-based bundles: PP-based bundles (log-likelihood = 289.91, *p* < 0.001***), “others” (log-likelihood = 139.77, *p* < 0.001***), NP-based bundles (log-likelihood = 82.52, *p* < 0.001***), infinitive construction (log-likelihood = 23.39, *p* < 0.001***), and anticipatory it structure (log-likelihood = 6.83, *p* < 0.01**). The most enormous difference exists in PP-based bundles. Almost half of the four-word bundles belong to this category, but only about 30% of five-word bundles were grouped into it. Another noticeable difference was found in NP-based bundles whose proportion in five-word bundles is roughly 10% higher than that in four-word bundles. Moreover, the category of “others” also reveals significant variances between the two: it merely makes up 9.0% of four-word bundles, but takes up 15.3% of five-word bundles. Furthermore, four-word bundles only have a higher proportion in PP-based bundles, whereas five-word bundles have a higher percentage in NP-based bundles, anticipatory it structure, infinitive construction, and “others.”

Four-and five-word bundles also significantly differ in all structural subcategories. The most obvious difference lies in “other NP” (log-likelihood = 368.60, *p* < 0.001***), and PP-based bundles beginning with in (log-likelihood = 354.08, *p* < 0.001***). The category of other NP constitutes 22.6% of five-word bundles, which is more than 2 times higher than that of four-word bundles. By contrast, the proportion of PP-based bundles beginning with in in four-word bundles is two times higher than that in five-word bundles. Substantial difference was also found in the category of other PP-based bundles (log-likelihood = 92.87, *p* < 0.001***). It comprises 11.2% of four-word bundles, yet only makes up 6.6% of five-word bundles. Another difference exists in PP-based bundles beginning with of whose proportion in four-word bundles is 2.3% higher than that in five-word bundles. Overall, there is a higher proportion of NP with of-phrase fragment, PP-based bundles beginning with in, PP-based bundles beginning with of, PP-based bundles beginning with on, and other PP-based bundles in four-word bundles, but a higher percentage of other NP and PP-based bundles beginning with at in five-word bundles.

From the perspective of function, significant variances were found in the distribution of text-oriented bundles (log-likelihood = 15.96, *p* < 0.001***), and participant-oriented bundles (log-likelihood = 221.33, *p* < 0.001***). The most noticeable difference was observed in participant-oriented bundles. Only 2.5% of four-word bundles fall into this category, whereas 7.4% of five-word bundles belong to it. What follows is text-oriented bundles which constitute 42.4 and 38.4% of four-and five-word bundles, respectively. Compared with four-word bundles, five-word bundles have a more even distribution of different bundles in that it has a lower proportion of research- and text-oriented bundles yet a higher percentage of participant-oriented ones.

### Comparison between three-and five-word bundles

There are significant variances between three-and five-word bundles in the distribution of anticipatory it structure (log-likelihood = 68.17, *p* < 0.001***), VP-based bundles (log-likelihood = 59.91, *p* < 0.001***), infinitive construction (log-likelihood = 53.50, *p* < 0.001***), and “others” (log-likelihood = 20.56, *p* < 0.001***). One striking difference lies in VP-based bundles which make up 4.7% of four-word bundles, almost twice of that of five-word bundles. Another lies in infinitive construction whose proportion in three-and five-word bundles is 2.6 and 4.4%, respectively. What is more, 12.9% of three-word bundles fall into the category of “others,” but 15.3% of five-word bundles belong to this category. In general, while three-word bundles contain a higher proportion of NP-based bundles, PP-based bundles, and VP-based bundles, five-word bundles have a higher percentage of anticipatory it structure, infinitive construction, and “others.”

Though significant variances were not found in the distribution of NP- and PP-based bundles, they were observed in their subcategories: PP-based bundles beginning with at (log-likelihood = 791.89, *p* < 0.001***), PP-based bundles beginning with in (log-likelihood = 242.60, *p* < 0.001***), PP-based bundles beginning with of (log-likelihood = 79.87, *p* < 0.001***), PP-based bundles beginning with on (log-likelihood = 57.86, *p* < 0.001***), other PP-based bundles (log-likelihood = 36.90, *p* < 0.001***), and NP with of-phrase fragment (log-likelihood = 15.35, *p* < 0.001***). One of the most striking differences is that PP-based bundles beginning with at merely accounts for 1.2% of three-word bundles, but constitute 8.3% of five-word bundles. Another difference lies in PP-based bundles beginning with in whose proportion in three-word bundles is at least twice of that in five-word bundles. Additionally, PP-based bundles beginning with of takes up 10.9% of three-word bundles, which is obviously higher than that (7.0%) in five-word bundles. Overall, there is a higher proportion of NP with of-phrase fragment, PP-based bundles beginning with in, and PP-based bundles beginning with of in three-word bundles, but a higher percentage of other NP, PP-based bundles beginning with on, PP-based bundles beginning with at, and other PP-based bundles in five-word bundles.

With regard to function, three-and five-word bundles differ significantly in terms of research-oriented bundles (log-likelihood = 71.39, *p* < 0.001***), text-oriented bundles (log-likelihood = 22.53, *p* < 0.001***), and participant-oriented bundles (log-likelihood = 371.89, *p* < 0.001***). The most noticeable variance was found in participant-oriented bundles. While merely 2.1% of three-word bundles belong to this category, 7.4% of five-word bundles fall into it. Meanwhile, there is also a considerable difference in research-oriented bundles whose proportion in three-and five-word bundles is 63.6 and 54.2%, respectively. Furthermore, text-oriented bundles take up 34.3% of three-word bundles, but 38.4% of five-word bundles. Overall, while three-word bundles are comprised of a higher proportion of research-oriented bundles, five-word bundles have a higher proportion of text- and participant-oriented bundles.

## Discussion

The comparisons between three-and four-word bundles, four-and five-word bundles, and three-and five-words in their structures and functions have revealed significant variances between bundles of various lengths. Firstly, three-word bundles make up a dominating proportion of the bundles, followed by four-word bundles, with five-word bundles making a particularly small percentage. It is not hard to find that frequency and bundle lengths are inversely related. This corresponds to the results in previous studies (Hyland, 2008b; Hong and Hua, 2018; Cui and Kim, 2021b).

Secondly, in general, greater differences were observed in main structural category between three-and four-word bundles than between four-and five-word bundles, and three-and five-word bundles. For instance, three-and four-word bundles differ significantly in all six main structural categories, namely, NP-based bundles, PP-based bundles, VP-based bundles, anticipatory it structure, infinitive construction, and “others.” Four-and five-word bundles differ in all main categories except VP-based bundles, while three-and five-word bundles are different in anticipatory it structure, VP-based bundles, infinitive construction, and “others.” Similar features can also be observed in functional distribution. There are significant variances between three-and four-word bundles in research-, text- and participant-oriented bundles. But for four-and five-word bundles, they only differ in terms of text- and participant-oriented bundles.

Thirdly, three-word bundles have the highest proportion of NP-based bundles (46.5%) and VP-based bundles (4.7%), while four-word bundles have the highest percentage of PP-based bundles (48.4%). In addition, five-word bundles have the highest percentage of anticipatory it structure, infinitive construction, and “others.” When it comes to function, the highest proportion of research-oriented bundles was observed in three-word bundles (63.6%), followed by four-word bundles (55.1%) and five-word bundles (54.2%). Additionally, the highest proportion of text-oriented bundles lies in four-word bundles (42.4%), followed by five-word bundles (38.4%), and three-word bundles (34.3%). And the highest percentage of participant-oriented bundles was found in five-word bundles (7.4%). What follow are four-word bundles (2.5%) and three-word bundles (2.1%).

One possible explanation for the differences between three-, four-, and five-word bundles could be related to the frequency of certain bundles. In other words, the rankings of bundles matter. For instance, among the 10 most frequent three-word bundles, four of them belong to the category of other NP. They are ‘english language learners’, ‘the united states’, ‘english as a’, and ‘a second language’. However, only one out of the 10 most frequent four-word bundles belongs to this group, which is ‘english as a second’. Considering that the frequency of top 10 bundles in the lists is considerably higher than that of the last 10 bundles. Hence this difference would inevitably exert significant influence on the total frequency of bundles that belong to this group. Another example is PP-based bundles beginning with of. There are 10 bundles in both the four-and five-word bundle lists that fall within this categorization, yet they take up 9.3 and 7.0% of the four-and five-word bundle lists, respectively. A closer look reveals that only two five-word bundles are the among the top 50 ones, whereas four four-word bundles are among the top 50 ones. In terms of function, among the top 10 three-word bundles, only three of them are text-oriented ones, which are ‘as well as’, ‘in order to’, and ‘in this study’. By contrast, this number is increased to five for four-word bundles. They are ‘on the other hand’, ‘as a result of’, ‘as well as the’, ‘the end of the’, and ‘at the end of’. This might explain the proportional variances of text-oriented bundles between three-and four-word bundles.

Another explanation is that though shorter bundles might form parts of longer ones, they could still differ enormously in terms of structure and function. For instance, three-word bundle ‘one of the’ is not only part of four-word bundles like ‘one of the most’, ‘is one of the’, and ‘was one of the’, but also part of five-word bundles like ‘one of the most important’ and ‘is one of the most’. Structurally, however, ‘one of the’, ‘one of the most’ and ‘one of the most important’ belong to the category of ‘others’, while ‘is one of the’, ‘was one of the’, ‘is one of the’ and ‘was one of the’ are VP-based bundles. Functionally, ‘one of the’, ‘is one of the’ and ‘was one of the’ are research-oriented bundles, yet ‘one of the most’, ‘one of the most important’ and ‘is one of the most’ are grouped into participant-oriented bundles. Another example is the three-word bundle ‘english language learners’. It comprises not only such four-word bundles as ‘of english language learners’, ‘english language learners in’ and ‘for english language learners’, but also five-word bundles like ‘of english language learners in’. But they differ greatly in their structures. Bundles like ‘of english language learners’ and ‘of english language learners in’ are categorized into PP-based bundles beginning with of, whereas ‘for english language learners’ is grouped into the category of other PP-based bundles. And ‘english language learners’ and ‘english language learners in’ belong to the category of other NP.

A closer look at the data suggests that bundles of different lengths differ in patterns that are used to realize the same function. On the one hand, three-word bundles mainly realize text orientation through using such patterns as NP with of-phrase fragment (e.g., a result of), PP-based bundles beginning with in (e.g., in addition to), and other PP-based bundles (e.g., because of the). Four-word bundles realize this function primarily by NP with of-phrase fragment (e.g., the role of the), PP-based bundles beginning with in (e.g., in the case of), PP-based bundles beginning with at (e.g., at the end of), and other NP (e.g., participants in this study). Five-word bundles realize it mainly by NP with of-phrase fragment (e.g., the results of the study), PP-based bundles beginning with at (e.g., at the beginning of the), PP-based bundles beginning with in (e.g., in the context of the), and other PP-based bundles (e.g., as a result of this). On the other, for three-word bundles, research orientation is mainly realized by four patterns: NP with of-phrase fragment (e.g., the use of), other NP (e.g., english language arts), PP-based bundles beginning with of (e.g., of the english), and PP-based bundles beginning with in (e.g., in the classroom). For four-word bundles, it is mainly by five patterns: NP with of-phrase fragment (e.g., the ministry of education), PP-based bundles beginning with of (e.g., of second language acquisition), PP-based bundles beginning with in (e.g., in the teaching of), other NP (e.g., the english as a), and other PP-based bundles (e.g., through the use of). For five-word bundles, it is primarily through five patterns: NP with of-phrase fragment (e.g., the ministry of education in), other NP (e.g., education in the united states), PP-based bundles beginning with of (e.g., of english language learners in), PP-based bundles beginning with in (e.g., in the teaching of writing), and other PP-based bundles (e.g., to speakers of other languages).

In this study, noun phrase-based and preposition phrase-based bundles are the dominating ones in three-, four-, and five-word bundles lists, which is in line with prior research (Hyland, 2008a; Hyland and Jiang, 2018; Shirazizadeh and Amirfazlian, 2021; Cui and Kim, 2021b). What is more, the following order can be found in the three bundle lists: research-oriented bundles make up a dominating proportion, followed by text-oriented bundles, with participant-oriented bundles making a small percentage. This is consistent with the findings of many previous studies (Hyland, 2008a; Hyland and Jiang, 2018; Shirazizadeh and Amirfazlian, 2021; Cui and Kim, 2021b).

The current study provides evidence that bundle lengths could not only influence the bundles one can get from a corpus, but also their structures and functions. It shows the potential influence of identification process-related variables on the structure and function of bundles. Along with previous studies which have demonstrated that bundles vary across registers (Biber and Barbieri, 2007), genres (Shirazizadeh and Amirfazlian, 2021), disciplines (Nekrasova-Beker and Becker, 2020), time (Hyland and Jiang, 2018), mother tongues (Pan et al., 2020), language proficiency (Chen and Baker, 2016), and stay-abroad experience (Edmonds and Gudmestad, 2021), this study provides further evidence that bundles also differ according to varying lengths.

## Conclusion

To summarize, the results of the study reveal that bundles of varying lengths differ significantly in terms of structure and function. Structurally, three-and four-word bundles differ in all six main categories. The most noticeable difference lies in PP-based bundles which comprise 32.7% of three-word bundles, but 48.4% of four-word bundles. Substantial differences were also found in all subtypes except NP with of-phrase fragment. As for four-and five- word bundles, significant variances lie in all main categories except VP-based bundles. One major difference is in PP-based bundles. It makes up almost half of four-word bundles, but only accounts for around 30% of five-word bundles. Considerable variances were also found in all subcategories. When it comes to three-and five-word bundles, significant differences lie in anticipatory it structure, VP-based bundles, infinitive construction, and “others.” Also, these two groups of bundles vary noticeably in six subcategories. In general, three-word bundles have the highest proportion of NP- and VP-based bundles, while four-word bundles contain the highest percentage of PP-based bundles. And five-word bundles are comprised of the highest proportion of anticipatory it structure, infinitive construction, and “others.”

Functionally, three-and four-word bundles differ substantially in all categories. The most considerable difference lies in text-oriented bundles which constitute 34.3% of three-word bundles, but 42.4% of four-word bundles. With regard to four-and five-word bundles, noticeable variances exist in text- and participant-oriented bundles. The most substantial difference was found in participant-oriented bundles. While only 2.5% of four-word bundles fall into this category, 7.4% of five-word bundles belong to it. As far as three-and five-word bundles’ concerned, significant differences lie in all categories. One considerable variance was found in participant-oriented bundles which merely make up 2.1% of three-word bundles, but take up 7.4% of five-word bundles. In general, three-, four-, and five-word bundles have the highest proportion of research-, text-, and participant-oriented bundles, respectively. Interestingly, three-, four-, and five-word bundles also differ in patterns that are used to perform the same functions.

The present study is one of the first to reveal the variances of bundles of different lengths in their structural and functional distribution, the results of which might have important implications for researchers. Considering the substantial variances that bundles of different lengths have demonstrated in structure and function, researchers are supposed to specify bundle lengths when making generalizations of their findings, and compare bundles of the same length when making comparisons between different studies. Notably, this study has the following limitations. Firstly, it only involved doctoral dissertations, which might limit our understanding of a whole picture because it is hard to tell whether we would get the exact same results if research articles, theses, and other forms of academic writings were also involved. Secondly, the study only analyzed the 100 most frequent three-, four-, and five-word bundles, respectively. Due to the relatively small number of bundles, we did not further divide the function of research-, text-, and participant-oriented bundles into subcategories. For future studies, it is suggested to compile a larger corpus of different types of academic writings and analyze a greater number of bundles, so that a more complete picture as to how bundles of different lengths differ in structure and function would be available.

## Data availability statement

The raw data supporting the conclusions of this article will be made available by the authors, without undue reservation.

## Ethics statement

Ethical review and approval was not required for the study on human participants in accordance with the local legislation and institutional requirements. Written informed consent was not provided because this study did not directly involve any participants.

## Author contributions

All authors listed have made a substantial, direct, and intellectual contribution to the work and approved it for publication.

## Conflict of interest

The authors declare that the research was conducted in the absence of any commercial or financial relationships that could be construed as a potential conflict of interest.

## Publisher’s note

All claims expressed in this article are solely those of the authors and do not necessarily represent those of their affiliated organizations, or those of the publisher, the editors and the reviewers. Any product that may be evaluated in this article, or claim that may be made by its manufacturer, is not guaranteed or endorsed by the publisher.
